# Is the association between optimistic cardiovascular risk perceptions and lower rates of cardiovascular disease mortality explained by biomarkers of systemic inflammation or endothelial function? A case-cohort study

**DOI:** 10.1186/1751-0759-4-11

**Published:** 2010-09-21

**Authors:** Robert Gramling, Kathi L Heffner, William MP Klein, Laura E Zajac, Mary Roberts, Charles B Eaton

**Affiliations:** 1 Department of Family Medicine, University of Rochester, 1381 South Avenue, Rochester, NY 14620, USA; 2 Department of Psychiatry, University of Rochester, 601 Elmwood Ave., Box PSYCH, Rochester, NY 14642, USA; 3 Division of Cancer Control and Population Sciences, National Cancer Institute, 6130 Executive Boulevard, Room 6134 Executive Plaza North, Rockville, MD 20852, USA; 4 Department of Psychology, University of Pittsburgh, 3425 Sennott Square, 210 S. Bouquet St., Pittsburgh, PA 15260, USA; 5 Center for Primary Care & Prevention, Memorial Hospital of Rhode Island 111 Brewster Street, Pawtucket, RI 02860, USA

## Abstract

**Background:**

More optimistic perceptions of cardiovascular disease risk are associated with substantively lower rates of cardiovascular death among men. It remains unknown whether this association represents causality (i.e. perception leads to actions/conditions that influence cardiovascular disease occurrence) or residual confounding by unmeasured factors that associate with risk perceptions and with physiological processes that promote cardiovascular disease (i.e. inflammation or endothelial dysfunction).

**Purpose:**

To evaluate whether previously unmeasured biological markers of inflammation or endothelial dysregulation confound the observed association between cardiovascular disease risk perceptions and cardiovascular disease outcomes;

**Methods:**

We conducted a nested case-cohort study among community-dwelling men from Southeastern New England (USA) who were interviewed between 1989 and 1990 as part of the Pawtucket Heart Health Program. We measured C-reactive protein (CRP) and Vascular Endothelial Growth Factor (VEGF) levels from stored sera for a random sample of the parent cohort (control sample, n = 127) and all cases of cardiovascular death observed through 2005 (case sample, n = 44). We evaluated potential confounding using stratified analyses and logistic regression modeling.

**Results:**

Optimistic ratings of risk associated with lower odds of dying from cardiovascular causes among men (OR = 0.39, 95% CI = 0.17, 0.91). Neither CRP nor VEGF confounded these findings.

**Conclusions:**

The strong cardio-protective association between optimistic ratings of cardiovascular disease risk and lower rates of cardiovascular mortality among men is not confounded by baseline biomarkers of systemic inflammation or endothelial dysfunction.

## Background

Optimistic perceptions of cardiovascular disease (CVD) risk are common [1-7] and often targeted by public health risk communication campaigns in hopes of changing CVD risk behavior (*i.e. *raising perception of CVD risk to motivate heart healthy actions)[7]. However, a growing body of work observes that optimistic perceptions of CVD risk predict lower rates of myocardial infarction [8-10], thus raising the question of whether seeking to overcome these "optimistic biases" will benefit or harm public health.

**Figure 1:**
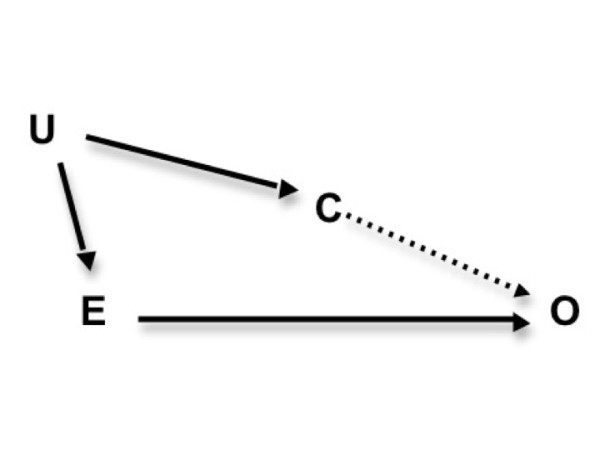
**Hypothetical sources of unmeasured confounding and adjustment for CRP and VEGF**. E = Comparative optimism in heart disease risk. O = CVD death. C = Systemic inflammation/endothelial dysfunction. U = Unmeasured potential confounder (*e.g. *personality, mood) A Directed Acyclic Graph (DAG) is a useful methodological tool for considering bias and confounding in complex associations. The DAG shown above in Figure 1 graphically represents how measures of systemic inflammation and endothelial dysfunction, **C**, relate to a set of plausible, yet unmeasured psychological confounders, **U **(*i.e. *mood, self-rated health, and personality) of the relation between comparatively optimistic ratings of CVD risk, **E**, and CVD death, **O**. Under hypothetical conditions where unmeasured psychological factors contributed to substantial residual confounding, adjusting for **C **(as this study does) blocks major physiological pathways that would be responsible for such residual confounding by **U**.

Considering plausible causal mechanisms *via *which optimistic biases might act to prevent heart disease is important; however, we must also thoroughly examine whether our prior observations of the CVD risk perception--outcome association are, in fact, *confounded *by unmeasured CVD risk factors. Risk perceptions are plausibly influenced by psychological conditions (*i.e. *personality, mood, coping style) that influence inflammatory status and endothelial reactivity. Since biomarkers of systemic inflammation (C-reactive protein) and endothelial dysregulation (Vascular Endothelial Growth Factor) also predict CVD events [11-16], these physiologic processes represent important pathways for potential confounding of the risk perception--CVD outcome association (see conceptual diagram in Figure 1). We hypothesize that controlling for markers of systemic inflammation and endothelial dysregulation will weaken earlier observations of a strong protective association between optimistic ratings of CVD risk and 15-year CVD mortality among community-dwelling men [9].

## Methods

### Overview

We conducted a case-cohort study nested within a community-based sample of men from southeastern New England (USA). A case-cohort study is an efficient epidemiologic design similar to a case-control study, except that the control group is chosen without regard to the outcome (i.e. a random sample of the base population), thus allowing comparison to multiple "case" groups (e.g. cardiovascular death, as in the current analysis). This strategy averts the time and financial cost of analyzing frozen sera from a full cohort.

All participants completed household interviews between 1990 and 1993. Serum samples were obtained during household interviews and frozen per standard protocol. Death record information was obtained from the National Death Index through December 2005 to identify cases. In depth description of this nested case-cohort study [16] and the parent cohort [9] from which participants were sampled has been published elsewhere.

### Participants and eligibility

All participants were enrolled during the post-intervention period of the Pawtucket Heart Health Program (PHHP), a controlled community intervention designed to lower prevalence of CVD risk factors [17]. One thousand one hundred seventy four men from the intervention and control cities who were between the ages of 40 and 74 years and had no personal history of myocardial infarction completed household interviews in the years immediately following the PHHP intervention (1989-1991). From this base population, sera were analyzed for all 44 cases of cardiovascular death identified through 2005 (see description below) and for a random sub-cohort of 127 men that was selected without regard to outcome. The random sub-cohort included 9 of the 44 cases.

### Exposure definition

Participants were asked at the beginning of the household interview, "Compared with persons of your own age and sex, how would you rate your risk of having a heart attack or stroke within the next 5 years?" Participants rated their CVD risk as "high", "average", "low", or "don't know". Endorsing one's risk to be lower than average we refer to as an optimistic rating.

### Case definition and selection

We obtained the date of death and underlying cause of death by linking personal identifiers (first name, middle initial, last name, sex, exact day/month/year of birth) for each participant to the Center for Disease Control National Death Index (NDI) for the years 1990-2005. Linkage and cause of death were established using NDI-standardized protocols. Cases were those participants whose primary or contributing cause of death was due to cardiovascular disease, as defined by International Classification of Disease (ICD) codes for coronary heart disease or stroke (ICD-9 codes: 410-414 and 430-438; ICD 10 codes: 120-125 and 160-169).

### Biomarker measurement

Using thawed sera, the concentrations of CRP and VEGF were measured using immunoturbidimetric and ELISA assays, respectively. Prior epidemiologic research has verified the validity of these samples and measurement procedures [16].

### Confounding and Effect Measure Modification

We considered the following as potential confounders or effect measure modifiers of the association between comparably optimistic self-rated CVD risk and CVD mortality: CRP, VEGF, age, race/ethnicity, income, education, foreign birth, city, Framingham Risk Score, total/HDL cholesterol ratio, hypertension, smoking status, body mass index, 1^st ^degree family history of early-onset coronary heart disease, current use of lipid or blood pressure lowering medications, current aspirin use, physical inactivity, alcohol, and psychotropic medications.

### Analyses

We evaluated the frequency and distribution of key study variables. For univariate comparisons shown in Table 1, we used the chi-square (categorical variables) and the Student's t-test (continuous variables) for determining statistical significance. Both VEGF and CRP demonstrated substantially skewed distributions with long right-sided tails. Thus, for all analyses considering these biomarkers as continuous variables, we perform natural logarithm transformations. Next, we evaluated potential confounder status for both CRP and VEGF by examining their association with optimistic ratings of risk in the random sub-cohort, since this represents the source population from which cases arise. Similarly, we examined whether CRP and VEGF were associated with CVD mortality, using the full case-cohort sample. We performed similar analyses for other potential confounding variables.

**Table 1:** Description of Sample by Case Status

	**No CVD death (n = 118)**	**CVD death (n = 44)**
	
Comparative optimists (%)*	36.4	18.2
City (%)		
Pawtucket	40.7	40.9
Control City	59.3	59.1
Race (%)		
African-American	10.2	4.6
Asian/Pac Island	1.7	4.6
Native American	0.0	2.3
White	88.1	88.6
Hispanic Ethnicity (%)	1.7	4.6
Age (%)*		
<45	21.2	6.8
45-64	61.9	52.3
65+	16.9	40.9
Education (%)		
<HS	49.2	45.5
HS	22.9	29.6
Some Coll	11.9	15.9
Coll Grad+	16.1	9.1
Income (%)		
<$10,000	10.9	17.5
$10,000-$19,999	27.3	42.5
$20,000-$29,999	22.7	20.0
$30,000-$39,999	10.9	7.5
$40,000-$49,999	8.2	2.5
$50,000+	20.0	10.0
Foreign Born (%)	36.4	27.3
Current Cigarette Smoker (%)	28.0	36.4
Physical Inactivity (%)	64.4	61.4
BMI - mean (SD)	27.7 (4.08)	28.0 (4.94)
Self-reported DM (%)*	5.9	22.7
Hypertension (%)*	50.0	77.3
TC - mean (SD)	227 (43.2)	228 (38.7)
HDL - mean (SD)**	45 (12.1)	41 (10.8)
C-reactive protein		
Mean (SD)**^¶^	0.45 (1.12)	1.36 (1.14)
Tertiles (%)*^‡^		
1	34.8	9.1
2	33.9	38.6
3	31.4	52.3
Vascular Endothelial Growth Factor		
Mean (SD)**^¶^	5.42 (0.74)	5.70 (0.57)
Tertiles (%)*^‡^		
1	34.2	11.6
2	33.3	30.2
3	32.5	58.1

*p < 0.05 on chi-square test; **p < 0.05 on Student's t-test; ¶ log transformed

‡ Tertiles defined by biomarker distribution among sub-cohort chosen without respect for outcome status [CRP: < 0.96 = T1, 0.96-2.82 = T2, >2.82 = T3; VEGF: <166 = T1, 166-299 = T2, >299 = T3]

Since more elaborate adjustment for potential social, biologic and behavioral confounders (propensity score analysis [18] and censoring of early follow-up time) did not improve upon age and Framingham Heart Score adjustment in the parent cohort [9], we limited adjustment methods here to multivariate logistic regression modeling.

We used SAS software (Cary, NC) for all statistical analyses. Where p-values are reported, we used a statistical significance threshold of 0.05.

The institutional research review boards at Memorial Hospital of Rhode Island and the University of Rochester School of Medicine and Dentistry approved this study.

## Results

Thirty one percent of the participants endorsed their risk of having a cardiovascular event in the next five years as lower than average for their age and sex (*i.e. *optimistic ratings). Forty-four participants died of cardiovascular causes during the 15-year surveillance period. Higher levels of CRP and VEGF were associated with higher risk of CVD mortality (see Table 1). Differences in log-transformed mean levels of VEGF (5.51 *versus *5.38; p = 0.37) and CRP (0.56 *versus *0.52; p = 0.83) for comparative optimists compared to others were small and statistically insignificant.

The association between comparative optimism and a lower probability of CVD death was strong (OR = 0.39, 95% CI = 0.17, 0.91). Adjusting for CRP alone, VEGF alone, or in combination with age, income and Framingham Risk Score did not substantially change these findings [see Table 2]. Similar results were observed for biomarker classification as a continuous (log-transformed) or categorical (tertile) variable.

**Table 2:** Crude and adjusted association between comparative optimism and CVD mortality

		**OR (95% CI)**			
**Comparative Optimism?**	**Cases/Non-cases**	**Model 1**	**Model 2**	**Model 3**	**Model 4**
		
Yes	8/43	0.39 (0.17, 0.91)	0.36 (0.14, 0.89)	0.33 (0.13, 0.81)	0.23 (0.07, 0.73)
No	36/75	referent	referent	referent	referent

Model 1: crude association between comparative optimism and CVD death

Model 2: adjusted for ln(CRP)

Model 3: adjusted for ln(VEGF)

Model 4: adjusted for age, Framingham Risk Score, per capita household income, ln(CRP) and ln(VEGF)

## Discussion and Conclusions

Following previous findings of an independent association between optimistic perceptions of CVD risk and substantially lower rates of CVD mortality among men, we evaluated whether biomarkers of stress (*i.e. *inflammation and endothelial dysregulation) would confound these provocative observations. We observed that controlling for baseline CRP and VEGF did not weaken the protective association between optimistic ratings of CVD risk and CVD mortality.

Psychosocial and behavioral processes related to self-enhancement and optimism might explain our findings. Believing that one is invulnerable from future problems may enhance motivation to pursue other activities that, in turn, buffer the effect of stress on CVD risk. In addition, people who perceive lower risk may also plan to take measures to reduce risk, making their perceptions accurate.

Another possible explanation is that optimistic ratings of CVD risk reflect an optimistic personality. Optimists seem to experience better health than non-optimists. Among cardiac patients, optimists show faster recovery from cardiac bypass surgery [19] and are less likely to be hospitalized 6 months post-surgery [20]. The beneficial health effects of optimism may arise from the different ways that optimists and pessimists cope with stress. Optimism is correlated with active and problem-focused coping strategies [21] that may lead to better health practices. Research has found that optimists do seem to be more effective in changing their own health behaviors and they report engaging in more healthy behaviors than do pessimists [22]. For example, when compared with pessimistic patients, optimistic cardiac patients were more successful in lowering their fat intake and getting more exercise [23]. However, prior work observes that the correlation between optimistic risk perceptions and global optimism is weak, ranging from *r *= 0.14 to *r *= 0.33 [24], thus suggesting that optimistic appraisal of one's CVD risk represents a sub-domain of optimism with potentially important cardio-protective implications.

Fiscella [25] raises important questions about whether optimistic ratings of health status--well known to predict survival [26]-- confound the cardio-protective association with optimistic self-ratings of CVD risk observed in this cohort. Health status was not measured, thus precluding us from directly addressing confounding (or interaction between optimistic ratings of health status and risk). However, optimistic self-rated health is associated with lower levels of systemic inflammation [27,28]. We observed no such relationship between optimistic ratings of CVD risk and systemic inflammation. Furthermore, controlling for systemic inflammation failed to weaken the observed cardio-protective effects of optimistic ratings of CVD risk. Thus, we conclude that unmeasured confounding by self-rated health status is unlikely to threaten the validity of the strong relationship observed in the present study.

Our findings do not rule out the possibility for systemic inflammation or endothelial dysregulation to represent a partial causal mechanism linking risk perceptions to CVD mortality. For example, if heightened awareness of CVD risk leads to fear-related behaviors and physiological responses that promote systemic inflammation or endothelial reactivity, downstream effects on CRP levels might be observed. We measured CRP and VEGF at baseline only. Although this provides strong evidence that these biomarker-related phenomena do not *confound *the association between comparative optimism and CVD mortality, longitudinal assessment is necessary to determine the degree to which *changes *in systemic inflammation or endothelial dysregulation represents a causal pathway linking CVD risk perceptions and CVD death.

A better understanding of the substantial cardio-protective association between optimistic risk perceptions and cardiovascular health is essential to guide the preventive applications for emerging technologies (e.g. advanced imaging, predictive genomics) that will shape our society's understanding of cardiovascular risk.

## Abbreviations

CRP: C-reactive protein; VEGF: vascular endothelial growth factor; ICD: International Classification of Disease; NDI: National Death Index

## Competing interests

The authors declare that they have no competing interests.

## Authors' contributions

All authors participated in the interpretation of these findings, contributed substantively to the writing of this manuscript and approved this submission. RG, MR, and CB participated in data collection and initial analyses. WK, LZ, and KH participated in subsequent revised analyses.
